# Spatial alignment of chemoarchitecture and resting-state functional connectivity predicts short term weight restoration in anorexia nervosa

**DOI:** 10.1038/s41398-026-03920-y

**Published:** 2026-03-06

**Authors:** Arne Doose, Livio Tarchi, Maria Seidel, Joseph A. King, Fabio Bernardoni, Inger Hellerhoff, Daniel Geisler, Katrin Gramatke, Giovanni Castellini, Valdo Ricca, Veit Roessner, Paul M. Thompson, Stefan Ehrlich

**Affiliations:** 1 Division of Psychological and Social Medicine and Developmental Neurosciences, Translational Developmental Neuroscience Section, Faculty of Medicine, Technische Universität Dresden, Dresden, Germany; 2 German Center for Child and Adolescent Health (DZKJ), partner site Dresden/Leipzig, Dresden, Germany; 3 Psychiatry Unit, Department of Health Sciences, University of Florence, Florence, Italy; 4 Department of Child and Adolescent Psychiatry, Medical Faculty, Technische Universität Dresden, Dresden, Germany; 5 Imaging Genetics Center, Stevens Neuroimaging & Informatics Institute, Keck School of Medicine, University of Southern California, Los Angeles, CA USA

**Keywords:** Predictive markers, Molecular neuroscience

## Abstract

Resting-state functional connectivity (rsFC) studies have revealed altered regional homogeneity (ReHo) and degree centrality (DC) in individuals with anorexia nervosa (AN) compared to healthy controls (HC), but the underlying mechanisms remain unclear. Here we explored the spatial alignment with neurotransmitter receptor and transporter densities (i.e., “chemoarchitecture”, based on “reference” PET studies) as a potential explanatory factor. We investigated rsFC alterations in acutely underweight patients with AN (*n* = 87) and age-matched HC (*n* = 87) cross-sectionally at admission and then again after successful weight-restoration treatment. Global ReHo and DC maps were associated with the spatial distribution of neurotransmitter receptors, transporters and/or metabolic glucose uptake. First, the correlation between rsFC alterations in AN and chemoarchitecture was evaluated at the group/timepoint-level. Second, individual-level correlations of neuroreceptor maps with rsFC alterations were calculated to test for possible associations with early weight restoration. The acute state of AN was characterized by higher DC (but not ReHo) in brain regions with a higher cortical density of vesicular acetylcholine transporter (VAChT), dopamine transporter (DAT) and serotonin transporter (SERT). Conversely, weight restoration was associated with normalization of DC, especially in areas with a higher DAT density. Importantly, individual-level spatial correlations between VAChT, DAT and SERT densities and DC alterations at admission significantly predicted early weight gain over first 90 days of treatment. These results suggest that neurochemical context may underlie functional brain alterations, providing a preliminary step toward identifying biological risk signatures. Replication with individualized PET data will be crucial to validate their potential utility for treatment stratification and personalization.

## Introduction

Anorexia nervosa (AN) is a severe eating disorder characterized by restrictive energy intake, low body weight, body image distortions, and fear of weight gain [1, 2]. Up to four out of every 100 women will be affected by AN at some time during their lifespan [3], with significant public health burden [4]. AN is among the leading causes of years lived with disability among female young adults [5], but there is no direct pharmacological intervention currently available for the disorder [6]. Evidence-based pharmacological treatment in AN is thus currently limited to the management of co-occurring symptoms [6], while many aspects of the biological mechanisms influencing the clinical presentation of AN remain unclear.

To potentially inform current clinical practice on biological mechanisms underlying AN, one line of research has focused on the use of neuroimaging techniques [7]. Structural and functional alterations have been frequently reported in AN in comparison to healthy controls (HC). Structural neuroimaging studies have shown a widespread reduction in gray matter (GM) during the acute phases of eating restriction, which potentially normalizes after weight rehabilitation [8, 9]. Findings from functional magnetic resonance imaging (fMRI), including those focusing on resting state functional connectivity (rsFC) have been more heterogeneous [10]. In rsFC, the temporal correlations of task-independent intrinsic activity of the brain is used to derive metrics of co-activation between predefined regions of interest or on a voxel-wise level across the whole brain [11]. The voxel-wise approach enables investigation without reliance on a priori hypotheses on regions of interest [12].

Regional homogeneity (ReHo) quantifies the degree to which a single voxel – in resting state fMRI – is functionally similar or ‘integrated’ into the function of its direct neighbors [13]. Functionally, elevated ReHo values are interpreted as greater local synchronization of neural activity, whereas reduced ReHo may indicate disrupted regional coordination within intrinsic brain networks. Studies investigating ReHo in AN indicated group differences compared to HC in several regions, including the dorsolateral prefrontal cortex, occipital cortex, and insula - areas that have been linked to cognitive control, visual processing and interoception [14–16]. Another measure, degree centrality (DC), can be used to describe global and local network properties of the observed intrinsic activity of the brain [12]. DC indicates the centrality (or relevance) of a given voxel to the transfer of information across the brain [17]. Previously, DC alterations in AN have been reported in the inferior frontal gyrus, and linked to cognitive control [18], while a recent study by our group showed a normalization of a globally increased DC signal after weight restoration [15].

A more detailed investigation of the neurochemical framework underlying these rsFC alterations may help to elucidate the relationship between functional alterations and possible dysfunctional neurotransmission in AN [19, 20]. Unlike rsFC studies, employing positron emission tomography (PET) allows to map the spatial distribution and density of molecular targets such as neurotransmitter and receptors. PET studies, particularly in individuals recovered from AN, have consistently reported alterations in both serotonin and dopamine systems. Findings include reduced 5-HT receptor binding and altered serotonin transporter function [21] linked to altered striatal D₂/D₃ receptor binding [22–24]. Previous radioligand tracing studies may be leveraged to enrich observed results of rsFC studies [25, 26] in AN. In other words, the spatial distribution of observed rsFC alterations can be compared to the cortical density of neurochemical features of interest - such as neurotransmitter receptors or neurotransmitters transporters. In the current work, we will refer to these features as the *chemoarchitecture* of the brain [27].

### Aims

The overall aim of this study was to investigate how rsFC alterations in AN relate to the brains chemoarchitecture, and whether these relationships influence treatment response.

More specifically, the primary aim of the study was to investigate the underlying neurobiological mechanisms of rsFC alterations (DC, ReHo) in AN relative to HC by examining their relationship with neurotransmitter receptor and transporter architecture (i.e., chemoarchitecture density maps - for acetylcholine, dopamine, glutamate and serotonin). Additionally, maps of brain metabolic demand, such as glucose uptake, were also considered. We hypothesized that rsFC alterations in AN would spatially align with the density distribution of neurotransmitter receptors/transporters and high metabolic demand.

Secondly, to assess whether the chemoarchitectural properties of the brain shape the normalization of rsFC alterations following weight restoration, we conducted the same analysis in a longitudinal design, contrasting acutely underweight patients with AN before and after intensive weight restoration treatment. We hypothesized that the partial normalization of rsFC alterations upon weight restoration would be shaped by the underlying chemoarchitectural features.

The third aim of the study was to determine whether the extent of alignment between the spatial distribution of rsFC alteration and chemoarchitecture at the individual level was predictive of early treatment success, defined as weight restoration trajectories at 30, 60 and 90 days. We hypothesized that the baseline alignment between resting-state functional connectivity (rsFC) alterations and chemoarchitecture maps would serve as a predictive indicator of treatment outcomes.

## Methods and materials

### Sample and participants

After quality control (described below) data from a total of 174 female participants (AN *n* = 87, age 12.1–24.4 years; HC *n* = 87) were included in the present study. The present study was conducted on the same sample as our previously published resting-state fMRI study in AN [15]. Participants with AN were first assessed within 96 h of admission in a specialized treatment program for eating disorders (timepoint one, TP1), then again after intensive weight restoration (≥12% body mass index (BMI) increase, as an indicator of adherence to the treatment program; timepoint two, TP2) [15]. As the duration of this second time point varies between participants (in order to reach the above-mentioned weight criteria), weight and height was also recorded at 30, 60 and 90 days from admission. Standardized anthropometric measurements were performed between 7 and 8 am after an overnight fast and immediately prior to scanning. The treatment program was predicated on a structured refeeding approach aimed at rapidly reversing undernutrition, restoring somatic stability, and supporting mental recovery. Additionally, patients had individualized and group-based cognitive-behavioral psychotherapy, tailored to individual needs.

AN diagnoses were confirmed using an adapted version of the Structured Interview for Anorexia and Bulimia Nervosa (SIAB-EX) [28]. Patients were diagnosed according to the Diagnostic and Statistical Manual of Mental Disorders, 5th edition [1] and had a BMI under 17.5 (or below the 10th percentile if younger than 18 years old at the time of enrollment). AN patients were excluded if they received psychotropic medications within 6 weeks prior to the study (except for Selective Serotonin Reuptake Inhibitors - SSRI - that were allowed in AN, *n* = 2). Comorbid psychiatric diagnoses were given by an experienced psychiatrist after consideration of the information collected from the clinical interviews, medical records, observations by the treatment team.

HC participants had to be of normal weight, eumenorrheic and with no current or history of mental or severe medical or neurological illness. HC participants were excluded if they had a lifetime BMI lower than 10th percentile (for participants younger than 18 years old), a BMI lower than 18.5 kg/m^2^, or were obese. Potential psychiatric illness in HC was assessed with the Mini International Neuropsychiatric Interview [29] and any lifetime diagnosis of an eating disorder was assessed using the SIAB-EX [28]. Both interviews were performed by trained master’s/doctoral level research assistants under the supervision of a board-certified child and adolescent psychiatrist.

In addition to the aforementioned exclusion criteria, participants of both groups were excluded if they had any current diagnosis or history of organic brain syndrome, dementia, schizophrenia, psychosis, or bipolar disorder.

All participants completed psychiatric screening instruments to facilitate a comparison of symptom severity. These instruments included the Eating Disorder Inventory–2 [30] and the Beck Depression Inventory–II [31]. BMI standard deviation scores (BMI-SDS) were computed to provide an age and sex corrected index [32].

The study protocols were in full accordance with the Declaration of Helsinki and approved by the local Institutional Review Board (Technische Universität Dresden - EK14012011), and all participants (and, if younger than 18 years old, their legal guardians) gave written informed consent.

### Data acquisition

Brain MR images were acquired between 8 and 9 a.m. using standard sequences with a 3 T MRI scanner (TIM Trio; Siemens, Erlangen, Germany). The MRI scanner was equipped with a 12-channel head coil. The T1-weighted structural brain scans were acquired with a rapid acquisition gradient echo (MP-RAGE) sequence: number of slices=176; repetition time TR = 1900 ms; echo time TE = 2.26 ms; flip angle FA = 9°; slice thickness=1 mm; voxel size=1 × 1 × 1 mm³; field of view FoV = 256 × 224 mm²; bandwidth=200 Hz/pixel). Functional images were acquired using a gradient-echo T2*-weighted echo planar imaging (EPI) with the following parameters: tilted 30° towards anterior/posterior commissure (AC-PC) line (to reduce signal dropout in orbitofrontal regions); number of volumes=190; number of slices=40; TR = 2200 ms; TE = 30 ms; FA = 75°; in-plane resolutio*n* = 3.4 mm; slice thickness = 2.4 mm (1 mm gap resulting in a voxel size of 3.4 × 3.4 × 2.4 mm³); FoV=220 × 220 mm²; bandwidth = 200 Hz/pixel. Participants were instructed to lie still with closed eyes and to stay awake during scanning.

### Preprocessing

Functional and structural images were processed using SPM12 toolbox (http://www.fil.ion.ucl.ac.uk/spm/), within a custom framework implemented by Nipype. The quality of the fMRI data was evaluated by visual inspection. Additionally, the quality of data was assessed using visual inspection and artifact detection tools (ART) [33] to identify volumes with intensity outliers (>3 SD from the mean of the time series) and excessive movement (at 2 thresholds: >2 mm and the more conservative >1 mm in any direction, based on framewise displacement). The standard procedure for automatic exclusion of a participant for this study was set to 25% (*n* = 48) of the collected resting state frames, i.e., if 48 or more frames were classified as either a motion (2 mm threshold) or an intensity outlier, the subject was excluded.

A sample-specific DARTEL template was created using structural images from all subjects [34]. The functional images were corrected for temporal slice-timing and motion simultaneously, using a 4D algorithm for realignment [35]. As was the case in Seidel et al. [15] no additional field map correction for susceptibility-induced distortions was applied. The realigned files were co-registered to the subject’s structural brain image. The EPI volumes were then normalized to Montreal Neurological Institute (MNI) space using the DARTEL template and corresponding flow field. Using the DPARSF toolbox [36], regression of nuisance covariates from 24-motion parameters, and physiological noise from white matter and cerebrospinal fluid was performed. Nuisance regression of white matter and cerebrospinal fluid signal were estimated via the CompCor method [37]. Preprocessing of T1-weighted structural brain scans and estimation of cortical gray matter thickness and subcortical gray matter volumes was carried out using FreeSurfer software (http://surfer.nmr.mgh.harvard.edu, version 7.1.1).

### Resting state functional connectivity

DC and ReHo values were calculated using DPARSF [36]. The DC of a voxel was determined by the number of significant Pearson correlations between its time series and each gray matter voxel (also known as binary DC). Only positive correlations above a threshold of r = 0.25 were considered, to exclude voxels with weak correlations that may be attributed to imaging noise or white matter. ReHo calculation was performed on a voxel-by-voxel basis by calculating the Kendall’s coefficient of concordance, which estimates similarity in the time series of a given voxel to its nearest 26 voxels, thus estimating the degree of regional homogeneity [38]. Prior to subsequent analyses, DC and ReHo maps were standardized into subject-level z-score maps. Smoothing was applied after calculation of each parameter with a Gaussian kernel of 6 mm at full width half maximum. A gray matter mask (obtained from the MNI template with a threshold of a probability higher than 0.3) was used to remove non−brain tissue in DC and ReHo maps.

In an exploratory analysis, to examine the potential dependence of rsFC measures on structural brain changes, the spatial correlation approach described below was applied using a group-level vertex-wise CT map as a reference feature map (Supplementary Methods 1).

### Cortical density of neurotransmitters and transporters

The python package *neuromaps* [25] is a collection of methods and maps which allows for comparison of empirically measured maps (e.g., rsFC) with a selection of reference feature maps (that is, spatial density of neurotransmitter receptors and transporters, based on radiotracer binding studies). Reference feature maps for chemoarchitecture were retrieved, across four major neurotransmitter receptor classes: acetylcholine, dopamine, glutamate, and serotonin [25, 39]. Cortical density maps of their vesicular transporters were also retrieved (vesicular acetylcholine transporter – VAChT; dopamine transporter – AT; serotonin transporter – SERT) [25, 39]. As no reference study was found on vesicular glutamate transporters, it was not possible at the current time to investigate this family of membrane-bound proteins [25, 39]. Reference feature maps were derived from a series of published studies with varying sample sizes. For a detailed description on reference feature maps, see eMethods 2 and Figure S4 in the Supplementary Materials and the original PET studies [39–46].

### Statistical analysis

#### Group contrasts

A general linear model was employed to calculate the statistical difference between AN and HC for vertex-wise DC, with age as a covariate (Supplementary Methods 3). The same modeling approach was applied for ReHo and CT. Cross-sectional differences between AN and HC in DC and ReHo z-maps were assessed using standard independent samples t-tests as implemented by SPM12.

#### Longitudinal contrasts

FreeSurfer’s general linear model was also employed to calculate the statistical difference between voxel-wise rsFC alterations of AN acquired at TP1 and TP2 (TP1 > TP2, Supplementary Methods 3). Longitudinal contrasts between AN_TP1 and AN_TP2 z-maps were assessed using standard paired samples t-tests as implemented by SPM12.

#### Spatial correlation of rsFC

To enrich rsFC alterations as observed in AN with reference feature maps of choice, the “neuromaps” collection of procedures was employed in order to transform data to the native space of choice (fsaverage). In brief, “neuromaps” uses standard technical developments [47–50] to perform the desired transformation between spaces of registration, such as between surfaces (i.e., fsaverage to fsLR), or from volumes to surfaces (i.e., MNI152 to fsaverage, converting the cortical voxels to vertices by accurate non-linear mapping of one onto the other).

In the present work, surface-to-surface (for GM contrast) and volume-to-surface (for DC and ReHo) transformations were used to convert contrast maps to the common space of choice (fsaverage). Once transformed to *fsaverage*, “neuromaps” was also used to estimate the vertex-wise spatial correlation (Pearson coefficient) between results (contrast map) and chemoarchitecture features, across the whole surface [25]. To assess the significance of such correlations, null models were then computed by rotating reference images in order to derive the null distribution of correlation coefficients (spatial nulls, *n* = 5000) [51]. From here on, we refer to these maps as maps of *spatially enriched group-level alterations*.

Individual-level alterations for rsFC were retrieved by subtracting the HC mean from individual-level results, in a voxel-wise manner. This subtracted image was then correlated using the same procedure as described above, thus obtaining a single individual-level correlation coefficient for each participant. Henceforth, we refer to these correlation coefficients as *spatially enriched individual-level alterations*.

#### Longitudinal weight restoration prediction

Spatially enriched individual-level alterations were evaluated for associations with BMI-SDS change relative to admission (TP1) at 30, 60 and 90 days after treatment initiation (*n* = 87, 84, 66; dropout due to physical illness (e.g. Covid-19), discharge or withdrawal of consent). This analysis was performed using Eq. 1.

1 $$BMI-S{\widehat{{DS}_{lon}}}_{gitudinal}={{\beta }_{0}+\beta }_{1}* {BMISDS}+{\beta }_{2}* {{SPFC}+\beta }_{3}* {{SPFC}* \beta }_{4}* {BMISDS}+{\beta }_{5}* {age}$$

where the SPFC term describes spatially enriched individual-level alterations in rsFC.

A prediction was not made for BMI-SDS at TP2, as the study design employed BMI increase as the defining characteristic for TP2, and the treatment duration differed between participants. Normality of residuals was confirmed via visual inspection, and influential cases were assessed using Cook’s distance (*D* > 0.05).

## Results

### Sample characteristics

HC and AN participants did not differ in age at their initial assessment (TP1), but differed in BMI-SDS (lower in AN), and psychiatric symptoms (higher eating psychopathology and depression in AN; Table 1). At follow-up (TP2, mean 86 ± 25 days), patients with AN showed increased BMI-SDS values in comparison to the initial assessment, and exhibited a reduction in psychiatric symptoms (both eating disorder related and depressive symptoms; Table 1). Patients with AN also showed substantial increases in weight at 30 (mean ΔBMI = 1.37(0.39), mean BMI = 16.11, *p* <0.001), 60 (mean ΔBMI = 2.94(0.67), mean BMI = 17.67, *p* <0.001) and 90 (mean ΔBMI = 4.19(0.87), mean BMI = 18.72, *p* <0.001) days after initiating treatment (Table 1).

**Table 1:** Sample Descriptives.

	AN_TP1	AN_TP2	HC	AN_TP1 vs HC Student’s T-test p-value	AN_TP1 vs AN_TP2 Student’s T-test p-value
	(*n* = 87)	(*n* = 87)	(*n* = 87)		
Age (years)	15.94 ± 2.58 [12.1–24.4]	16.17 ± 2.58	16.08 ± 2.59 [12.1–24.6]	0.721	/
BMI	14.7 ± 1.3	18.7 ± 1.1	20.4 ± 2.3	<0.001	<0.001
BMI-SDS	−3.12 ± 1.08	−0.75 ± 0.61	−0.1 ± 0.71	<0.001	<0.001
BMI-SDS after 30 days	−2,15 ± 0,87	/	/	/	/
BMI-SDS after 60 days	−1,25 ± 0,71	/	/	/	/
BMI-SDS after 90 days	−0,73 ± 0,60	/	/	/	/
Duration of current episode (months)	10.43 ± 12.34	/	/	/	/
EDI-2 total	204.88 ± 42.65	186.1 ± 45.81	142.27 ± 26.83	<0.001	<0.001
BDI-II	21.71 ± 10.72	12.82 ± 10.16	5.07 ± 5.13	<0.001	<0.001

Mean value ± standard deviation (SD) for each variable and study group are shown. Time between AN_TP1 and AN TP2 [ ≥ 14% BMI increase, mean 27.9% ± 9.2]: mean=86 days; standard deviation=25 days. Nine patients were formally diagnosed with comorbid psychiatric disorders at AN_TP1 (Five major depressive disorder, three illness anxiety/somatic symptom disorder, one obsessive compulsive disorder, one post-traumatic stress disorder, one tic/Tourette diagnosis, one personality disorder).

A*N* anorexia nervosa, *HC* healthy controls.

Two participants with AN were excluded based on visual inspection of their fMRI images, along with their age- and sex-matched controls. None of the participants had to be excluded due to motion/intensity outliers.

### Resting state functional connectivity group-level alterations

**Fig. 1:**
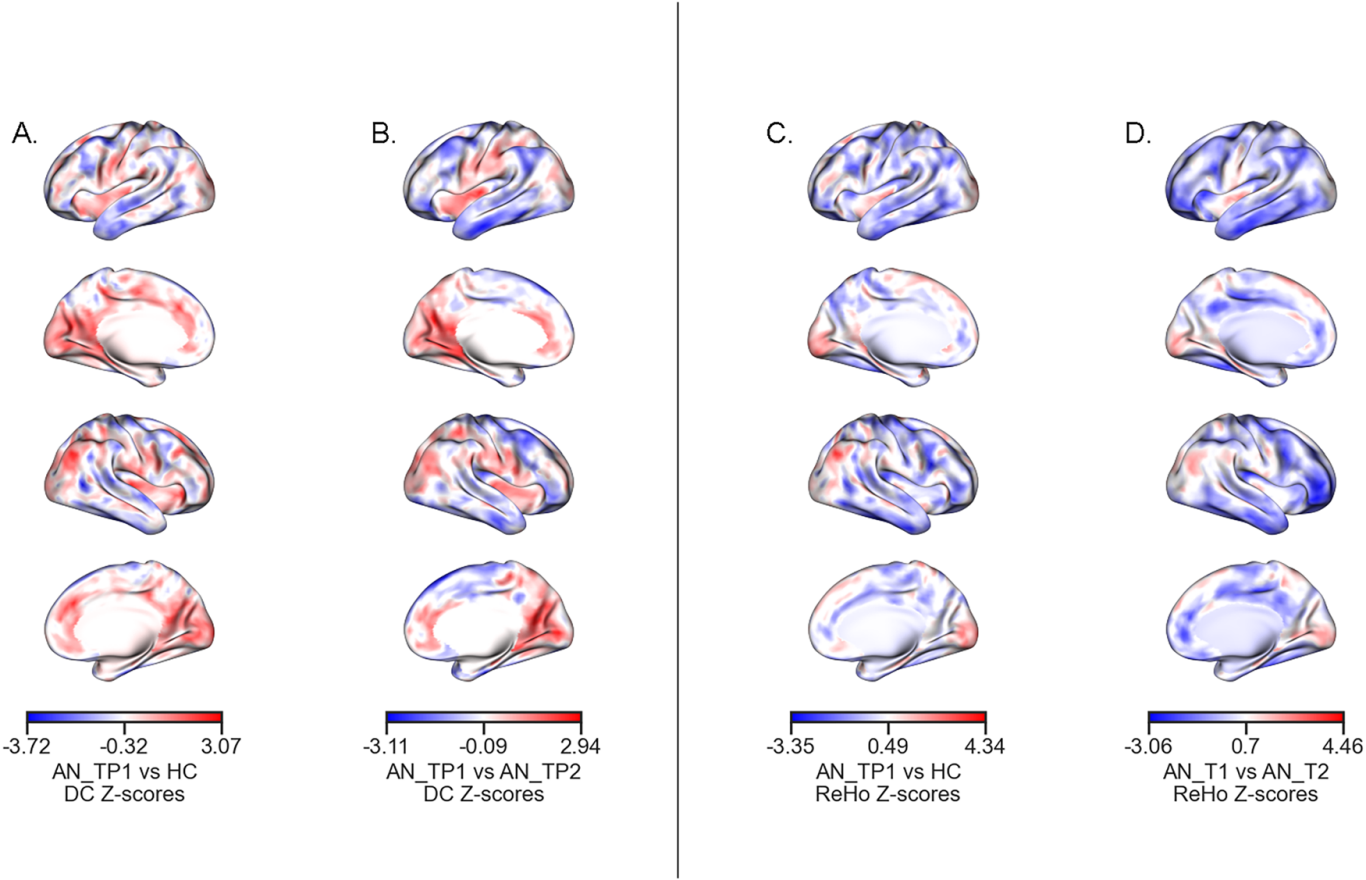
Group-level contrast of voxelwise resting state functional connectivity (rsFC) alterations in AN. **A** cross-sectional AN_TP1 vs HC, degree centrality (DC); (**B**) longitudinal AN_TP1 vs AN_TP2, DC; (**C**) cross-sectional AN_TP1 vs HC, regional homogeneity (ReHo); (**D**) longitudinal AN_TP1 vs AN_TP2, ReHo. A/C: in blue, higher values in HC. In red, higher in AN_TP1. B/D: in blue, higher values in AN_TP2. In red, higher in AN_TP1. Results are unthresholded. Abbreviations: AN_TP1 = acute AN at baseline = TP1, AN_TP2 = AN after weight-restoration at TP2.

DC was predominantly elevated in individuals with AN in comparison to HC within medial structures (paracingulate gyrus, cingulate cortex, medial occipital lobes, superior temporal gyrus, insula), and mostly decreased within lateral structures (caudal middle frontal gyrus, middle and inferior temporal gyrus, angular gyrus - panel A, Fig. 1). Longitudinal comparison of DC values between AN_TP1 and AN_TP2 showed a similar pattern with mostly increased DC in medial structures and mostly decreased DC in lateral structures at TP1 compared to TP2 (panel B, Fig. 1). Remarkably, the longitudinal contrast of AN_TP1 vs AN_TP2 was significantly and positively correlated with the group contrast of AN_TP1 vs HC, indicating that longitudinal changes were strongest in areas with initial cross-sectional changes (panel A-B, Fig. 1; Pearson *r* = 0.64, *p* < 0.001).

ReHo was mostly globally reduced in AN_TP1 in comparison to HC (panel C, Fig. 1), with a similar longitudinal pattern of lower ReHo in AN_TP1 compared to AN_TP2 (panel D, Fig. 1). Similar to DC, the group contrast of AN_TP1 vs HC was significantly and positively correlated with the group contrast of AN_TP1 vs AN_TP2 (panel C-D, Fig. 1; Pearson *r* = 0.56, *p* = 0.001).

Cross-sectional alterations of rsFC were also significantly and positively correlated with each other (DC and ReHo, AN_TP1 vs HC, Pearson *r* = 0.66, *p* < 0.001).

### Spatially enriched group-level alterations

**Fig. 2:**
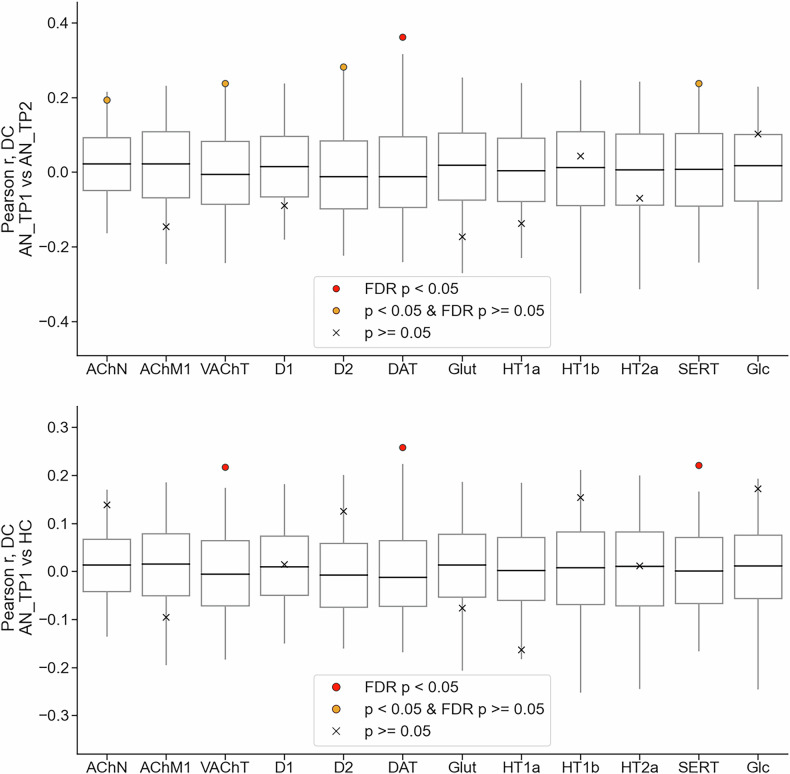
Spatial enrichment of group-level degree centrality alterations in AN. Boxplots represent the correlation coefficient for rotated images (5.000 permutations), in order to represent 95% confidence intervals of null distributions [51]. Upper panel: DC group difference of AN_TP1 vs HC. Lower panel: DC group difference of AN_TP1 vs AN_TP2. Empirical results are represented by a red point if statistically significant. DC degree centrality.

With regard to the primary aim of the study, cross-sectional rsFC differences between AN_TP1 and HC were analyzed in relation to the underlying chemoarchitecture of the brain. DC alterations during acute AN were significantly and positively correlated with the spatial distribution and density of all neurotransmitter transporters (*VAChT*: Pearson *r* = 0.22, *p* = 0.010; *DAT*: Pearson *r* = 0.26, *p* = 0.005; *SERT:* Pearson *r* = 0.22, *p* = 0.002; panel A, Fig. 2), but not with the neurotransmitter receptors or glucose metabolism. All DC neurotransmitter transporter results survived FDR correction for multiple comparisons (*VAChT*: adjusted-*p* = 0.039; *DAT*: adjusted-*p* = 0.031; *SERT:* adjusted-*p* = 0.029). By contrast, acute alterations of ReHo were not significantly associated with chemoarchitecture features or glucose metabolism (with a single significant correlation, HT1a, Pearson r = −0.18, *p* = 0.024, which did not survive FDR correction for multiple comparisons; Supplementary Materials Figure S1).

For our second aim, the hypothesized alignment of rsFC normalization with chemoarchitecture maps were investigated. As ReHo was not significantly associated with any chemoarchitectural feature at the cross-sectional level, only DC results were considered. The longitudinal contrast AN_TP1 vs AN_TP2 was positively and significantly associated with DAT (TP1 > TP2, Pearson *r* = 0.36, *p* = 0.004, FDR adjusted-p = 0.048; panel B, Fig. 2).

In summary, areas with a higher cortical density of VAChT, DAT and SERT showed higher DC in AN_TP1. Subsequently, areas with a higher cortical density of DAT were associated with DC decreases between AN_TP1 and AN_TP2.

### Spatially enriched individual-level alterations

With regard to our third aim, the investigation of the predictive value of individual-level alignment for early treatment outcome, the analyses were only conducted for chemo-architectural features that were significant at the group level (VAChT, DAT, SERT). As expected [52, 53], a lower BMI-SDS at AN_TP1 predicted a lower BMI-SDS at 90 days. Furthermore, the aforementioned typical chemo-architectural patterns observed in acute patients with AN (AN_TP1) as compared to healthy controls – i.e. higher spatially enriched individual-level rsFC alterations in regions with high VAChT, DAT and SERT density - were also predictive of lower BMI-SDS at 90 days, beyond the effect of BMI-SDS and age at baseline alone. This means that AN participants who were characterized by these typical spatial patterns of DC alterations at TP1, also showed lower weight restoration after 90 days. In addition, a significant interaction was observed between the typical “chemo-architectural” patterns of DC alterations and BMI-SDS at baseline (Table 2), facilitating the negative effect of lower BMI-SDS at baseline. In other words, a low BMI-SDS at baseline would predict an even lower BMI-SDS at 90 days if the individual patient exhibited the typical (i.e. as reported for the group-level analysis) chemo-architectural patterns of DC alterations. See Table 2 for further details.

**Table 2:** Prediction of BMI-SDS at 90 days, using spatially enriched individual-level alterations.

Model	Term	Beta	Beta standardized	*T*-value	*p*-value (FDR corrected)
Model 1	Age	−0.054	−0.219	−2.394	0.022
	BMI-SDS	0.402	0.745	7.994	0.003
	VAChT_DC	−2.934	−0.755	−2.642	0.012
	BMI-SDS *VAChT_DC	−0.903	−0.781	−2.679	0.012
Model 2	Age	−0.066	−0.270	−2.883	0.009
	BMI-SDS	0.372	0.690	7. 520	0.003
	DAT_DC	−1.968	−0.653	2.264	0.027
	BMI-SDS * DAT_DC	−0.656	−0.775	−2.665	0.012
Model 3	Age	−0.069	−0.282	3.235	0.005
	BMI-SDS	0.437	0.809	8.460	0.003
	SERT_DC	−4.146	−0.730	−2.895	0.009
	BMI-SDS * SERT_DC	−1.523	−0.917	3.509	0.003

Linear regression model predicting BMI-SDS, after 90 days of intensive treatment. All terms survived FDR correction (*p* < 0.05).

*SERT* serotonin transporter, *DAT* dopamine transporter, *VAChT* vesicular acetylcholine transporter.

All main effects (of the chemo-architectural patterns) and interaction terms survived FDR correction for multiple comparison. See Supplementary Materials Figure S2 for a graphical representation of results. No significant effect was observed at 30 or 60 days (Supplementary fig. S2).

### Sensitivity analyses

Sensitivity analyses for subtype and psychiatric comorbidity confirmed the results of the prediction models (Supplementary Results 1). The analysis of the spatial alignment of acute alterations in voxelwise rsFC and vertexwise CT revealed no significant alignment of DC or ReHo group-level alterations and CT (Supplementary Results 2).

## Discussion

The present study sought to investigate the relationship between rsFC alterations in AN and the brain’s chemoarchitecture and whether these relationships are associated with normalization after weight restoration and predictive of early treatment response. In summary, our results show that brain regions with a higher cortical density of VAChT, DAT and SERT, including but not limited to the striatum, medial prefrontal, insular, and anterior cingulate cortices, are characterized by higher DC (but not ReHo) in the acute state of AN compared to HC. During subsequent weight restoration, areas that were observed to have a higher cortical density of DAT were associated with a significant decrease in DC. Thus, our results indicate a substantial overlap between the underlying chemoarchitecture and brain areas affected by voxel-wise rsFC alterations in AN and, in part its subsequent treatment-related normalization. With regard to our third aim, and of particular clinical relevance, the individual associations of VAChT, DAT and SERT density with DC alterations were predictive of short-term weight-restoration, which has been described as a crucial precursor to treatment success [52, 53]. Notably, the direction of these individual effects was the same as that observed at the group level and the chemo-architectural were patterns consistent. Our results can be interpreted as preliminary evidence for a biomarker of short-term treatment success in AN patients. This biomarker is defined as a specific signature of AN-typical cross-sectional alterations in rsFC, which, if replicated, may potentially aid in the prognostic prediction of weight restoration trajectories at the individual level, and may therefore provide a foundation for future stratification and personalization of treatment approaches for AN.

Overall, and as reported in Seidel et al. [15], DC in AN at TP1 in comparison to HC was mostly increased in medial structures and mostly decreased in lateral structures, whereas ReHo was globally reduced. The positive correlations between higher DC and higher density of VAChT, DAT and SERT suggest that voxelwise rsFC alterations in the acute state and the observed subsequent partial normalization with weight restoration are, at least in part, influenced by the underlying neurotransmitter system and its spatial distribution. Prior studies suggest a direct effect of alterations in the neurotransmitter system on rsFC based on DC, as well as other graph-theoretical measures [54]. In particular, experimental modulation of the dopaminergic system through L-dopa has been demonstrated to reduce the overall communication efficiency and the organization into subnetworks of the functional connectome [55]. Similarly, serotonergic modulation with SSRIs has been shown to reduce the tendency of brain regions to form local clusters, alter the balance between local specialization and global integration (often called small-world properties), and decrease global average DC [56, 57].While the neurophysiological interpretation of alterations in DC and ReHo is complex [58], it can be assumed that neurotransmitter systems play a key role in neuronal excitability and therefore in the generation of the BOLD signal and its spontaneous fluctuations [59].

Given the widespread and substantial structural brain changes in acute AN [8, 9], the question arises whether and to what extent measures of voxel-wise connectivity such as DC and ReHo are affected by these changes. Previous research on the relationship between functional connectivity and the underlying GM structure has yielded inconclusive results [60, 61]. Our findings indicate that DC alterations do not appear to be driven by CT alterations, as no significant alignment was found between group-wise alterations in DC and CT. Previously reported higher CT reductions in regions with gene expression profiles of S1 pyramidal cells and oligodendrocytes suggest a link between CT alterations in AN and metabolic demand [8]. However, our results showed no correlation between rsFC measures and the spatial distribution of metabolic demand.

Longitudinally, we found that the normalization of DC in patients following weight restoration showed a significant alignment with DAT regions compared to the acute state of AN. In other words, the normalization of DC was higher in regions characterized by a high DAT density, and vice versa. Alterations in dopamine networks in AN have been linked to reinforcement learning processes associated with restrictive (eating) behavior in animal studies [62–64]. To date, no PET studies have examined DAT binding in AN. However, dopamine receptor binding has been demonstrated to be elevated in individuals with AN [22, 23]. A decrease in DC in DAT-associated regions with weight restoration, as shown in our data may indicate that the functional pattern captured by DC becomes less closely aligned with the dopaminergic architecture, even though some resting-state abnormalities persist after short-term weight restoration [15].

Importantly, we found that individual-level DC alterations in areas expressing VAChT, DAT and SERT predict early weight gain during intensive treatment after 90 days. Notably, the direction of the individual-level effect on early weight gain was aligned with the effect observed at the group level, i.e. a more “AN-typical” chemoarchitectural pattern was associated with less early weight gain. Higher early weight gain itself has been described as a predictor of a favorable long-term outcome [52, 53, 65]. Thus, our finding provides preliminary support for the idea that resting-state connectivity, when contextualized by neurotransmitter distribution, could carry prognostic information. Given the complexity and multitude of the transmitter systems involved, any discussion of mechanisms remains necessarily speculative. Nevertheless, the roles of dopamine, serotonin, and acetylcholine in reward valuation, motivation, and affect regulation are well documented, and disruptions in their associated circuits may help explain why stronger alignment with “AN-typical” chemoarchitecture predicted poorer early weight gain. Of note, the models of DC maps enriched with VAChT, DAT or SERT did not predict BMI-SDS after 30 and 60 days. One potential explanation for this may have been somatic factors such as refeeding-related edema that are a common unwanted effects early in treatment [66] which can bias weight measurements. Taken together, the results of the current study could potentially be relevant for personalized medicine approaches in the treatment of AN. Indeed, the identification of a biomarker for short-term treatment success in AN could facilitate the more efficient allocation of scarce healthcare resources by indicating which patients may require more intensive interventions (e.g., in terms of closely supervised meals, additional nutritional support or preferential inpatient vs. day time hospital treatment setting).

### Limitations

Although this study has numerous strengths, the results must be interpreted in the light of the following limitations. While the chemoarchitecture of reference feature maps, including neurotransmitter receptors, transporters, and glucose metabolism was derived from normative PET maps originally estimated using data primarily from the adult general population, our sample consisted of mostly adolescent female healthy participants as well as AN patients, potentially biasing the current results. Nevertheless, the only PET study on neurotransmitter transporters reported no group differences between participants recovered from AN in the SERT binding potential compared to HC [67], suggesting similar SERT distributions in AN. Future studies would benefit from combined rsfMRI–PET designs or large-scale, multisite samples to generate age- and diagnosis-specific chemoarchitectural maps. Furthermore long-term follow-up designs are needed to clarify whether chemoarchitectural alignment at admission becomes predictive of long term clinical outcome.

## Conclusions

The current study highlights the interplay between voxelwise rsFC alterations and chemoarchitecture in AN. We demonstrated that brain regions with a higher cortical density of VAChT, DAT and SERT exhibit higher DC in the acute state of AN. Conversely, areas with a higher cortical density of VAChT, DAT and SERT showed a decrease in DC after short-term weight restoration therapy, which may hint at state-related processes during acute underweight that normalize with treatment. Furthermore, the same patterns observed in AN at the group level, i.e. higher DC in regions with high density of VAChT, DAT and SERT and vice versa, were at the individual level predictive of lower early weight restoration. These findings offer preliminary insight into a potential biological signature of AN that may be linked to clinical outcomes. They may support future efforts toward stratification and personalization of treatment in AN. To illustrate, they may assist in identifying patients who could benefit from step-down or day-treatment after inpatient care, as opposed to those suitable for outpatient follow-up treatment. Nevertheless, the results must be regarded as hypothesis-generating, and their replication in larger clinical samples, preferably incorporating individualized PET data, will be essential to establish their robustness and translational relevance.

## Supplementary information

Supplemental material
