# Supplementary material for: Spatial alignment of chemoarchitecture and resting-state functional connectivity predicts short term weight restoration in anorexia nervosa
Source: Transl Psychiatry. 2026 Mar 6;16:138. doi: 10.1038/s41398-026-03920-y (PMC12982752; doi:10.1038/s41398-026-03920-y)
Supplement: Supplementary file 1 — Supplemental material [file 41398_2026_3920_MOESM1_ESM.docx]

# Supplementary Materials

**Methods 1 – Supplementary cortical thickness analysis**

To examine the potential dependence of rsFC measures on structural brain changes, the spatial correlation approach described below was applied using a group-level vertex-wise CT map as a reference feature map. Surface reconstruction was first performed for each hemisphere separately, including tessellation of the gray matter-white matter boundary, automated topology correction, and surface deformation following intensity gradients to optimally place the gray-white and gray-cerebrospinal fluid borders at the location where the greatest shift in intensity defines the transition to the other tissue class. The quality of the surface reconstruction and segmentation was assured by visual inspection by a trained examiner with the support of quality assurance tools implemented in FreeSurfer (https://surfer.nmr.mgh.harvard.edu/fswiki/QATools) and exploratory analysis of the parcellation and segmentation statistics for outliers as in our previous studies (Seidel et al., 2019).

**Methods 2 - Description of Feature Maps**

The AChN receptor density map was estimated using positron emission tomography (PET) radioligand [(18)F]flubatine in 16 participants (Hillmer et al., 2016). The spatial density of AChM1receptors was assessed using PET radiotracer 11C-LSN3172176 in six healthy participants (Naganawa et al., 2020). VAChT surface density map was estimated using PET tracer binding (SUVR) measures of 4 participants (Hansen et al., 2022). The D1 receptor surface density map was estimated using D1R-selective radiotracer [^11^C]SCH23390 in PET scans of thirteen healthy volunteers (Kaller et al., 2017). The spatial density of D2 receptors on the cortical surface was assessed by [^18^F]Fallypride binding at PET among 49 healthy participants (Jaworska et al., 2020). The transporter density map for DAT were measured through DAT-SPECT (Dukart et al., 2018). Glut density maps were estimated using high resolution [11C]ABP688 PET scans which were acquired in 74 healthy participants (Smart et al., 2019). The expression of serotonin receptors (HT1a, HT1b, HT2a) was estimated by [^11^C]CUMI-101, [^11^C]AZ10419369, and [^11^C]Cimbi-36 binding at PET, among healthy participants (8, 36 and 29 volunteers, respectively) (Beliveau et al., 2017). SERT distribution was assessed by PET tracer binding of [^11^C]MADAM among six young male subjects (age range 21-35 years old) and 10 older individuals (8 males and 2 females, age range 51-67 years old) (Fazio et al., 2016). Glucose metabolism (Glc) was evaluated using [18F]-labeled fluorodeoxyglucose and assessing task-induced aerobic glycolysis in 33 healthy volunteers (Vaishnavi et al., 2010).

Please refer to the original studies for further information about included reference maps.

**Methods 3 - Equation used to calculate the statistical difference between AN and HC for vertex-wise DC**

Equation 1: $\hat{DC}= \beta_{0}+\beta_{1} *age+\beta_{2} *group$

In the longitudinal analysis, the second coefficient in Equation 1 (β_2*group) represented the longitudinal contrast.

**Results 1**

As part of our secondary analyses, the prediction models were repeated in (1) the subgroup of patients diagnosed with the restrictive subtype of AN (*n =* 79), and (2) after excluding AN participants with psychiatric comorbidity (Depression *n =* 5, OCD = 1, Anxiety Disorder = 3); Again – and replicating the findings in our main analysis – if an individual exhibited a high DC in cortical regions characterized by a high density of SERT or VAChT receptors, a lower BMI-SDS at 90 days could be predicted (beyond the effects of BMI-SDS and age at baseline alone; see Supplementary Materials Table S1).

**Results 2**

To examine a possible dependence on structural brain changes, the spatial alignment of acute alterations in voxelwise rsFC and vertexwise CT was evaluated. No significant alignment was found between acute alterations in DC and CT (group contrasts AN_TP1 vs HC, *r* = 0.00, *p* = .972). Similarly, no significant alignment was found between acute alterations in ReHo and CT (group contrasts AN_TP1 vs HC, *r* = 0.01, *p* = .850).

| **Table S1 - Prediction of BMI-SDS at 90 days, using spatially enriched individual-level alterations limited to participants with AN diagnosis of the restrictive subtype.** | | | | | | |  |
| --- | --- | --- | --- | --- | --- | --- | --- |
| Model | Term | Beta | 2.5% | 97. 5% | T-value | p-value | p-value  (FDR corrected)v |
| Model 1 | Age | -0.056 | -0.104 | -0.007 | -2.311 | 0.024 | 0.036 |
|  | BMI-SDS | 0.391 | 0.279 | 0.503 | 6.982 | <0.001 | 0.003 |
|  | VAChT_DC | -2.543 | -4.974 | -0.112 | -2.095 | 0.041 | 0.045 |
|  | BMI-SDS * VAChT_DC | -0.797 | -1.556 | -0.039 | -2.105 | 0.040 | 0.045 |
| Model 2 | Age | -0.068 | -0.118 | -0.019 | -2.751 | 0.008 | 0.016 |
|  | BMI-SDS | 0.362 | 0.255 | 0.469 | 6.787 | <0.001 | 0.003 |
|  | DAT_DC | -1.677 | -3.555 | 0.200 | -1.789 | 0.079 ° | 0.079 |
|  | BMI-SDS * DAT_DC | -0.573 | -1.120 | -0.026 | -2.097 | 0.040 ° | 0.045 |
| Model 3 | Age | -0.077 | -0.122 | -0.031 | -3.343 | 0.001 | 0.003 |
|  | BMI-SDS | 0.432 | 0.317 | 0.546 | 7.560 | <0.001 | 0.003 |
|  | SERT_DC | -3.851 | -6.908 | -0.794 | -2.522 | 0.014 | 0.024 |
|  | BMI-SDS * SERT_DC | -1.496 | -2.454 | -0.539 | -3.130 | 0.003 | 0.007 |
| Legend: ° did not survive FDR correction for multiple comparison (p < 0.05) | | | | | | |  |

| **Table S2 - Prediction of BMI-SDS at 90 days, using spatially enriched individual-level alterations excluding participants with current psychiatric disorders.** | | | | | | |  |
| --- | --- | --- | --- | --- | --- | --- | --- |
| Model | Term | Beta | 2.5% | 97. 5% | T-value | p-value | p-value  (FDR corrected) |
| Model 1 | Age | -0.051 | -0.122 | 0.021 | -1.426 | 0.16° | 0.16 |
|  | BMI-SDS | 0.399 | 0.286 | 0.512 | 7.072 | <0.001 | 0.004 |
|  | VAChT_DC | -2.919 | -5.339 | -0.449 | -2.420 | 0.019 | 0.033 |
|  | BMI-SDS * VAChT_DC | -0.845 | -1.622 | -0.128 | -2.350 | 0.023 | 0.034 |
| Model 2 | Age | -0.069 | -0.142 | 0.004 | -1.907 | 0.05° | 0.055 |
|  | BMI-SDS | 0.334 | 0.252 | 0.476 | 6.523 | <0.001 | 0.004 |
|  | DAT_DC | -2.108 | -4.009 | -0.206 | -2.224 | 0.027 | 0.036 |
|  | BMI-SDS * DAT_DC | -0.692 | -1.243 | -0.141 | -2.519 | 0.010 | 0.020 |
| Model 3 | Age | -0.068 | -0.135 | 0.000 | -2.004 | 0.05 ° | 0.055 |
|  | BMI-SDS | 0.432 | 0.309 | 0.537 | 7.435 | <0.001 | 0.004 |
|  | SERT_DC | -4.420 | -7.647 | -1.194 | -2.749 | 0.008 | 0.019 |
|  | BMI-SDS * SERT_DC | -1.620 | -2.635 | -0.605 | -3.203 | 0.002 | 0.006 |
| Legend: ° did not survive FDR correction for multiple comparison (p < 0.05) | | | | | | |  |

**Figure S1**

**
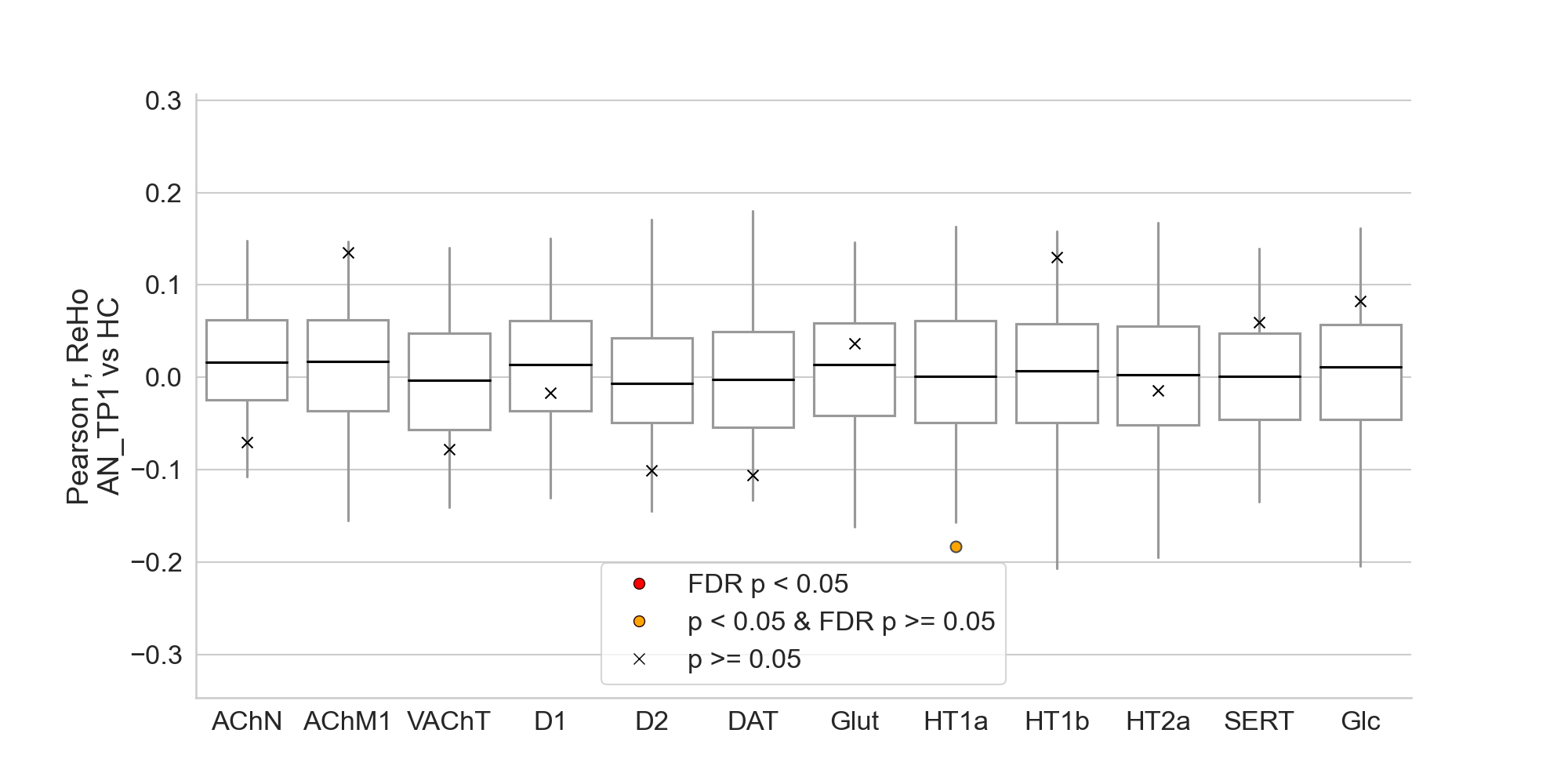
**

**Figure S1 - Spatial enrichment of group-level regional homogeneity alterations in AN.** Boxplots represent the correlation coefficient for rotated images (5.000 permutations), in order to represent 95% confidence intervals of null distributions. Empirical results are represented by a red point if statistically significant. ReHo = regional homogeneity


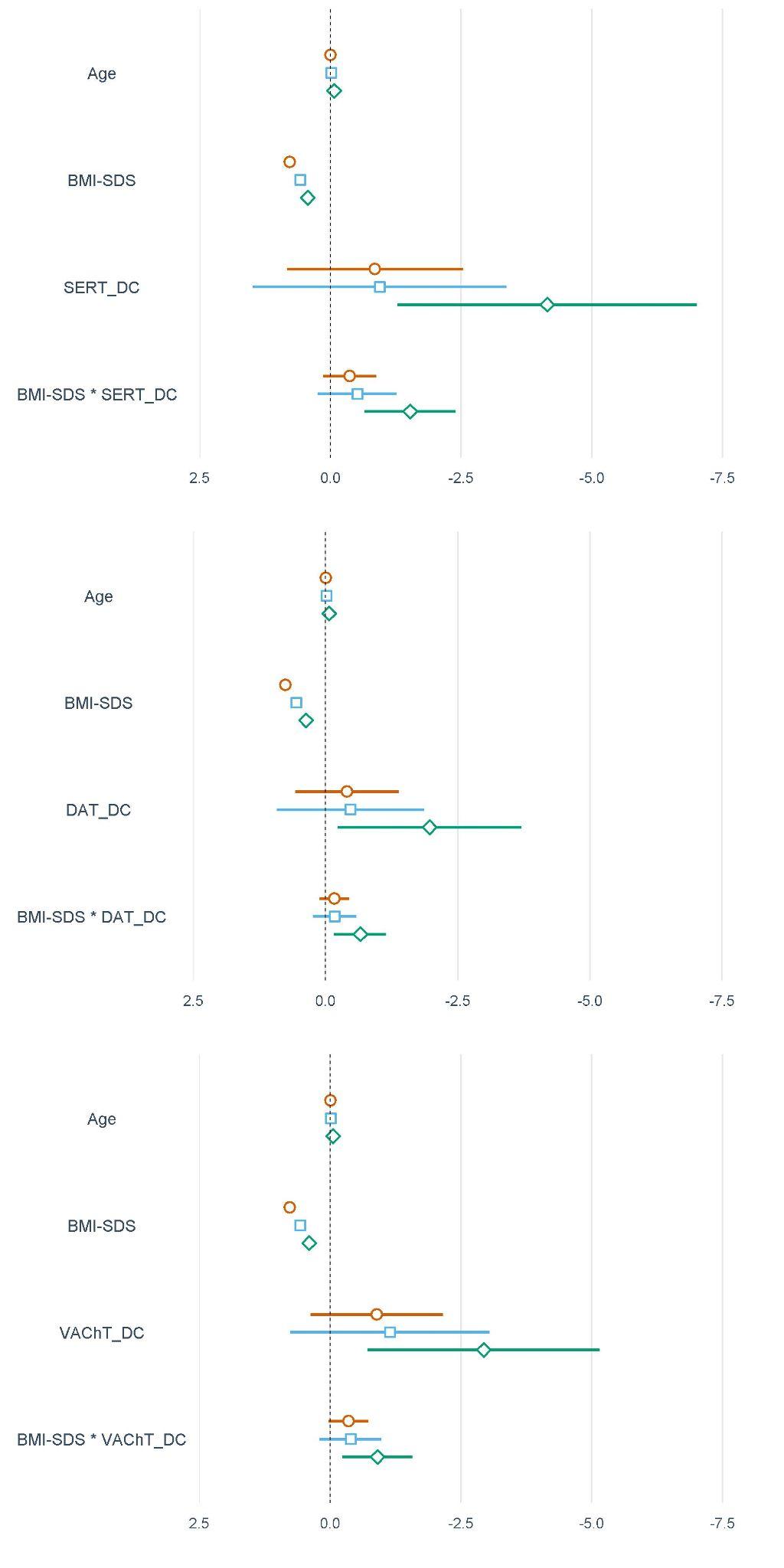


**Figure S2 - Spatially enriched individual-level alterations were predictive of BMI-SDS at 90 days, beyond BMI-SDS and age at baseline alone.** No significant effect was observed at 30 or 60 days. In orange, the model for BMI-SDS at 30 days. In cyan, the model for BMI-SDS at 60 days. In green, the model for BMI-SDS at 90 days. Legend: SERT = serotonin transporter, DAT = dopamine transporter, VAChT = vesicular acetylcholine transporter


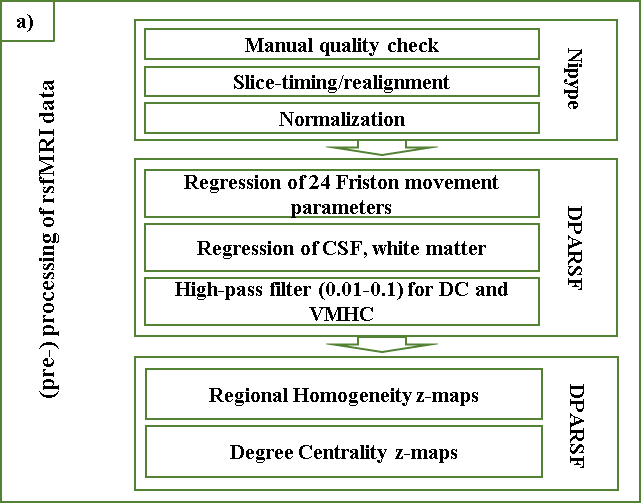
**
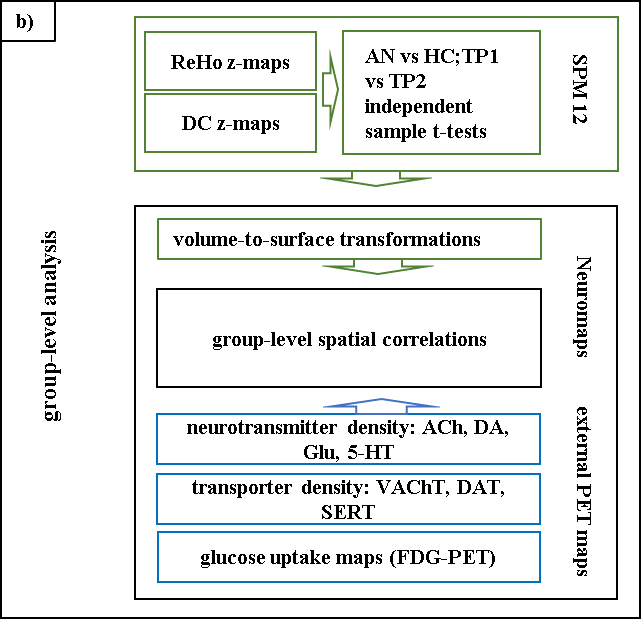
**
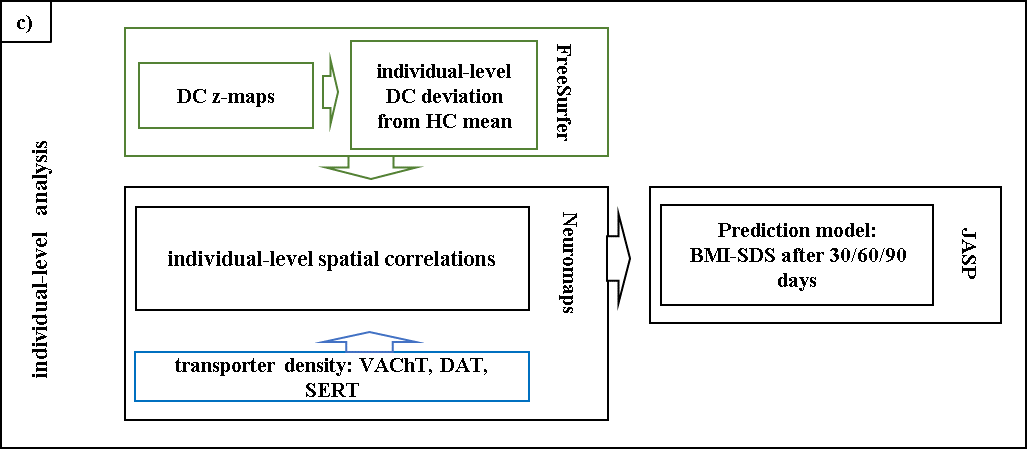


**Supplementary Figure S3: (a) Preprocessing, (b) group-level analysis flow and (c) individual-level analysis flow with corresponding software.** In green: analysis flow of rsfMRI data. In blue: external PET data as part of the *neuromaps* toolbox. Abbreviations: AN = anorexia nervosa, HC = healthy controls, TP1 = timepoint 1 (admission), TP2 = timepoint 2 (partial weight restoration), BMI = body mass index, BMI-SDS = body mass index standard deviation score, QC = quality control, rsfMRI = resting-state functional magnetic resonance imaging, rsFC = resting-state functional connectivity, DPARSF = MATLAB toolbox called Data Processing Assistant for Resting-State fMRI (Yan & Zang, 2010), Nipype = neuroimaging data processing framework, SPM 12 = Statistical Parametric Mapping (*SPM12*, 2020), Neuromaps = python toolbox to compare brainmaps (Markello et al., 2022), FreeSurfer = image analysis suite for brain surface data (Fischl et al., 1999), JASP = statistical software (JASP, 2019), DC = degree centrality, ReHo = regional homogeneity, CT = cortical thickness, VAChT = vesicular acetylcholine transporter, DAT = dopamine transporter, SERT = serotonin transporter, FDG-PET = fluorodeoxyglucose positron emission tomography.


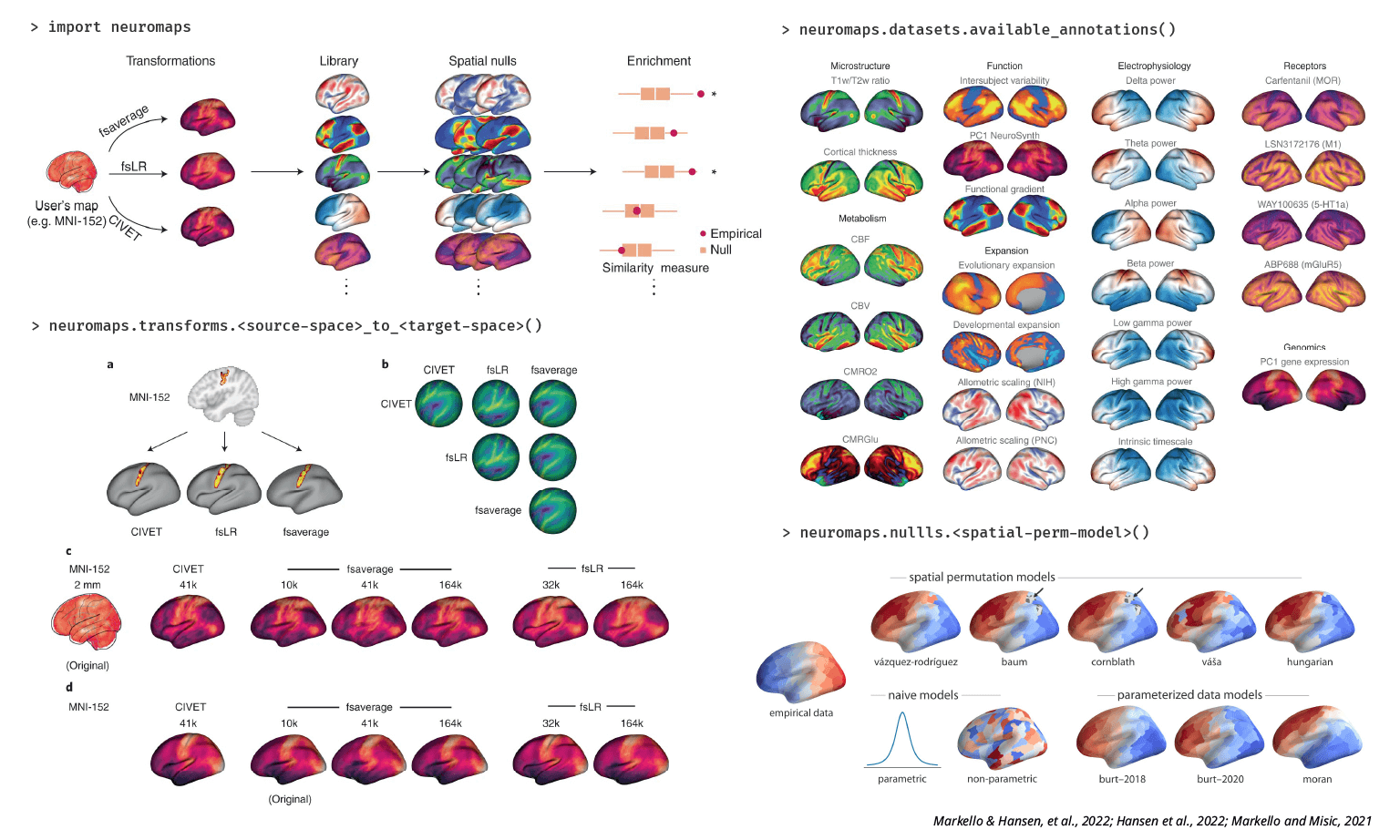


**Figure S4. Details of the features of the neuromaps toolbox** (copied from Markello & Hansen et al. (2022), Hansen et al. (2022) Markello & Misic et al. (2021)), available at <https://github.com/netneurolab/neuromaps>. This image is licensed under the Creative Commons Attribution-NonCommercial-ShareAlike 4.0 International License (https://creativecommons.org/licenses/by-nc-sa/4.0/).

**References**

Beliveau, V., Ganz, M., Feng, L., Ozenne, B., Højgaard, L., Fisher, P. M., Svarer, C., Greve, D. N., & Knudsen, G. M. (2017). A High-Resolution In Vivo Atlas of the Human Brain’s Serotonin System. *Journal of Neuroscience*, *37*(1), 120–128. https://doi.org/10.1523/JNEUROSCI.2830-16.2016

Dukart, J., Holiga, Š., Chatham, C., Hawkins, P., Forsyth, A., McMillan, R., Myers, J., Lingford-Hughes, A. R., Nutt, D. J., Merlo-Pich, E., Risterucci, C., Boak, L., Umbricht, D., Schobel, S., Liu, T., Mehta, M. A., Zelaya, F. O., Williams, S. C., Brown, G., … Sambataro, F. (2018). Cerebral blood flow predicts differential neurotransmitter activity. *Scientific Reports*, *8*(1), 4074. https://doi.org/10.1038/s41598-018-22444-0

Fazio, P., Schain, M., Varnäs, K., Halldin, C., Farde, L., & Varrone, A. (2016). Mapping the distribution of serotonin transporter in the human brainstem with high-resolution PET: Validation using postmortem autoradiography data. *NeuroImage*, *133*, 313–320. https://doi.org/10.1016/j.neuroimage.2016.03.019

Fischl, B., Sereno, M. I., & Dale, A. M. (1999). Cortical surface-based analysis: II: inflation, flattening, and a surface-based coordinate system. *Neuroimage*, *9*(2), 195–207.

Hansen, J. Y., Shafiei, G., Markello, R. D., Smart, K., Cox, S. M. L., Nørgaard, M., Beliveau, V., Wu, Y., Gallezot, J.-D., Aumont, É., Servaes, S., Scala, S. G., DuBois, J. M., Wainstein, G., Bezgin, G., Funck, T., Schmitz, T. W., Spreng, R. N., Galovic, M., … Misic, B. (2022). Mapping neurotransmitter systems to the structural and functional organization of the human neocortex. *Nature Neuroscience*, *25*(11), Article 11. https://doi.org/10.1038/s41593-022-01186-3

Hillmer, A. T., Esterlis, I., Gallezot, J. D., Bois, F., Zheng, M. Q., Nabulsi, N., Lin, S. F., Papke, R. L., Huang, Y., Sabri, O., Carson, R. E., & Cosgrove, K. P. (2016). Imaging of cerebral α4β2* nicotinic acetylcholine receptors with (−)-[18F]Flubatine PET: Implementation of bolus plus constant infusion and sensitivity to acetylcholine in human brain. *NeuroImage*, *141*, 71–80. https://doi.org/10.1016/j.neuroimage.2016.07.026

JASP. (2019). *JASP* (Version 0.11.1) [Computer software]. https://jasp-stats.org/download/

Jaworska, N., Cox, S. M. L., Tippler, M., Castellanos-Ryan, N., Benkelfat, C., Parent, S., Dagher, A., Vitaro, F., Boivin, M., Pihl, R. O., Côté, S. M., Tremblay, R. E., Séguin, J. R., & Leyton, M. (2020). Extra-striatal D2/3 receptor availability in youth at risk for addiction. *Neuropsychopharmacology*, *45*(9), Article 9. https://doi.org/10.1038/s41386-020-0662-7

Kaller, S., Rullmann, M., Patt, M., Becker, G.-A., Luthardt, J., Girbardt, J., Meyer, P. M., Werner, P., Barthel, H., Bresch, A., Fritz, T. H., Hesse, S., & Sabri, O. (2017). Test-retest measurements of dopamine D1-type receptors using simultaneous PET/MRI imaging. *European Journal of Nuclear Medicine and Molecular Imaging*, *44*(6), 1025–1032. https://doi.org/10.1007/s00259-017-3645-0

Markello, R. D., Hansen, J. Y., Liu, Z.-Q., Bazinet, V., Shafiei, G., Suárez, L. E., Blostein, N., Seidlitz, J., Baillet, S., Satterthwaite, T. D., Chakravarty, M. M., Raznahan, A., & Misic, B. (2022). neuromaps: Structural and functional interpretation of brain maps. *Nature Methods*, *19*(11), 1472–1479. https://doi.org/10.1038/s41592-022-01625-w

Naganawa, M., Nabulsi, N. B., Henry, S., Matuskey, D., Lin, S., Slieker, L., Schwarz, A. J., Kant, N., Jesudason, C., Ruley, K., Navarro, A., Gao, H., Ropchan, J., Labaree, D., Carson, R. E., & Huang, Y. (2020). First in Human Assessment of the Novel M1 Muscarinic Acetylcholine Receptor PET Radiotracer 11C-LSN3172176. *Journal of Nuclear Medicine*. https://doi.org/10.2967/jnumed.120.246967

Seidel, M., Borchardt, V., Geisler, D., King, J. A., Boehm, I., Pauligk, S., Bernardoni, F., Biemann, R., Roessner, V., Walter, M., & Ehrlich, S. (2019). Abnormal Spontaneous Regional Brain Activity in Young Patients With Anorexia Nervosa. *Journal of the American Academy of Child & Adolescent Psychiatry*, *58*(11), 1104–1114. https://doi.org/10.1016/j.jaac.2019.01.011

Smart, K., Cox, S. M. L., Scala, S. G., Tippler, M., Jaworska, N., Boivin, M., Séguin, J. R., Benkelfat, C., & Leyton, M. (2019). Sex differences in [11C]ABP688 binding: A positron emission tomography study of mGlu5 receptors. *European Journal of Nuclear Medicine and Molecular Imaging*, *46*(5), 1179–1183. https://doi.org/10.1007/s00259-018-4252-4

*SPM12*. (2020). [Computer software]. Wellcome Trust Centre for Neuroimaging. https://www.fil.ion.ucl.ac.uk/spm/

Vaishnavi, S. N., Vlassenko, A. G., Rundle, M. M., Snyder, A. Z., Mintun, M. A., & Raichle, M. E. (2010). Regional aerobic glycolysis in the human brain. *Proceedings of the National Academy of Sciences*, *107*(41), 17757–17762. https://doi.org/10.1073/pnas.1010459107

Yan, C.-G., & Zang, Y.-F. (2010). DPARSF: A MATLAB Toolbox for “Pipeline” Data Analysis of Resting-State fMRI. *Frontiers in Systems Neuroscience*, *4*, 13. https://doi.org/10.3389/fnsys.2010.00013
